# Persistent low-Level viremia in persons living with HIV undertreatment: An unresolved status

**DOI:** 10.1080/21505594.2021.2004743

**Published:** 2021-12-07

**Authors:** Celia Crespo-Bermejo, Eva Ramírez de Arellano, Violeta Lara-Aguilar, Daniel Valle-Millares, Mª Luisa Gómez-Lus, Ricardo Madrid, Luz Martín-Carbonero, Verónica Briz

**Affiliations:** a Laboratory of Reference and Research on Viral Hepatitis, National Center of Microbiology, Institute of Health Carlos Iii, Majadahonda, Madrid, Spain; b Departamento de Medicina- Área de Microbiología. Facultad de Medicina. Universidad Complutense, Madrid, Spain; c Parque Científico de Madrid, Campus de Cantoblanco, Madrid, Spain; d Department of Genetics, Physiology and Microbiology. Faculty of Biology, Complutense University of Madrid, Madrid, Spain; e Unidad de Vih. Servicio de Medicina Interna. Hospital Universitario La Paz. Instituto de Investigación Sanitaria Hospital de La Paz (Idipaz), Madrid, Spain

**Keywords:** HIV, pLLV, ART adherence, VF, reservoir, clonal expansion, immune activation, mortality, aids events and nades

## Abstract

Antiretroviral therapy (ART) allows suppressed viremia to reach less than 50 copies/mL in most treated persons living with HIV (PLWH). However, the existence of PLWH that show events of persistent low-level viremia (pLLV) between 50 and 1000 copies/mL and with different virological consequences have been observed. PLLV has been associated with higher virological failure (VF), viral genotype resistance, adherence difficulties and AIDS events. Moreover, some reports show that pLLV status can lead to residual immune activation and inflammation, with an increased risk of immunovirological failure and a pro-inflammatory cytokine level which can lead to a higher occurrence of non-AIDS defining events (NADEs) and other adverse clinical outcomes. Until now, however, published data have shown controversial results that hinder understanding of the true cause(s) and origin(s) of this phenomenon. Molecular mechanisms related to viral reservoir size and clonal expansion have been suggested as the possible origin of pLLV. This review aims to assess recent findings to provide a global view of the role of pLLV in PLWH and the impact this status may cause on the clinical progression of these patients.

## Introduction

Human Immunodeficiency virus (HIV) viremia is related to increased AIDS events and death [1]. Nonetheless, the use of antiretroviral therapy (ART) reduces morbidity and mortality in persons living with HIV (PLWH) [2,3]. According to international HIV treatment guidelines, the main objective of ART is to achieve and maintain undetectable viral load (VL) over time, decrease HIV transmission, and avoid the emergence of drug resistance [4,5]. The establishment of a cutoff point of VL at which a patient can develop clinical events is thus essential. However, two different thresholds have been identified according to different international guidelines: 200 copies/mL [Department of Health and Human Services’ (DHHS, USA)] [4] and 50 copies/ml (European AIDS Clinical Society) [5]. Current treatments enable levels of virologic suppression below the detection sensitivity of many standard assays [6]. Nevertheless, ART does not eradicate the virus and residual viremia (≈1-10 copies/mL) have been found in a large number of patients after years of highly suppressive therapy [6–9].

Transient episodes of detectable viremia (blips) have been described in around 1/5 of HIV-infected patients undergoing suppressive treatment, whereas 4–10% of PLWH show persistent events of low-level viremia (≈50–500 copies/mL) [10–12]. Clinical consequences vary considerably according to the type of incomplete virologic suppression in question. For instance, no association has been found between blips and a greater risk of virologic and immunological failure [12–14], while persistent low-level viremia (pLLV) has been associated with the emergence of drug resistance [11,15–17], virological failure [10,14,18,19] and alteration of immune status [20–22].

Today, the role of pLLV in PLWH remains unclear and a lot of unresolved questions complicate management of these individuals. The aim of this paper is to review the possible causes and origins of pLLV in PLWH and the impact that pLLV may have on the clinical progression of these patients according to recent findings.

## Persistent low-level viremia: Definition and clinical implications

The scientific community has reached no definition regarding pLLV patients. In general, treated patients with HIV persistently presenting viremia from 50 to 1000 copies/ml have been considered pLLV patients (Table 1). However, clinical implications differ when considering patients with pLLV between 50 and 200 copies/ml [23–25] and patients with higher viremias (up to 400–500 [26–28] or up to 1000 copies/mL [11,29]).

**Table 1.** Association between persistent low-level viremia in PLWH under ART (current studies)

								ART regimen in pLLV		
Year	STUDY	N	Follow-up (years)	pLLV patients (n)	pLLV definition (cp/mL)	VF definition (cp/mL)	VF (YES/NO)	NRTIs + PI/r	NRTIs + NNRTI/II	Others
2015	**[**27**]**	17,902	2.7	624	VL = 50–199	VL ≥ 200	Yes, between 200–499	351	273	-
				482	VL = 200–499			237	244	-
2015	**[**24**]**	2374	11	205	VL = 50–199	VL ≥200	Yes	135	70	-
2015	**[**23**]**	2276	1	127	VL ≤ 50	VL ≥ 200	No	114	108	-
				95	VL = 51–199					-
2017	**[**32**]**	1015	20	716	VL < 50	VL ≥ 1000	Yes, between 200–999	UNS	UNS	-
				46	VL = 50–199					
				52	VL = 200–999					
2018	**[**30**]**	5986	11	237	VL = 50–199	VL ≥ 500	Yes, between 200–499	100	124	13
				168	VL = 200–499			82	69	17
2018	**[**31**]**	70,930	9	9901	VL = 51–199	VL ≥ 1000	Yes	UNS	UNS	-
				3358	VL = 200–399					
				3609	VL = 400–999					
2019	**[**28**]**	2795	10	152	VL = 51–200	VL ≥ 200	Yes	UNS	UNS	-
				110	VL = 201–500					
2019	**[**25**]**	2006	21.8	374	iLLV: VL = 50–199 on < 25% of measurements	VL ≥ 200* or > 1000**	Yes, in iLLV patients.	UNS	UNS	-
				152	pLLV: VL = 50–199 on ≥ 25% of measurements					-
2020	**[**33**]**	508	8	86	VL = 50–1000	VL > 1000	Yes	5	81	-

Notes: pLLV: Persistent low-level viremia, VF: Virological failure, VL: Viral load, cp/mL: Copies/mL; ART: Antiretroviral treatment; iLLV: Intermittent LLV; UNS: Unspecified. NRTI: Nucleoside reverse transcriptase inhibitors; PI: Protease inhibitors; NNRTI: Non-nucleoside reverse transcriptase inhibitors; II: Integrase inhibitors. * On two consecutive measurements. ** During ART and six months after initiation of ART.

An association between pLLV and a higher risk of **virological failure** (VF) has been previously observed (Table 1). Patients with pLLV >200 copies/mL experience 3–4 times the risk of developing VF than patients with ≤200 copies/ml [27,28,30], and up to 5 times the risk in patients with LLV between 400 and 999 cp/mL [31]. By contrast, the association between a pLLV below 200 cp/ml (50–200 cp/mL) and VF is unclear. While some studies have found an increased risk of VF only in patients with LLV between 201 and 500 cp/mL and not in patients with ≤200 copies/mL compared to undetectable HIV patients [30,32], others, such as the recent study by Joya and colleagues (2019) [25], suggest that patients with lower persistent viremia (≤200 copies/mL) may experience a risk of VF HIV that is 3.46 times higher than that of suppressed patients. Moreover, in a more sensitive analyses, patients with LLV between 201 and 500 cp/mL showed that the risk of virologic failure may become significant depending on whether patients are ART-naïve or ART-experienced (aHR 1.61 (0.45, 1.11), p > 0.05; aHR 3.50 (1.25, 9.81), p < 0.05; respectively).

Low adherence, usually defined as taking less than 80% of prescribed drugs, and genotypic resistance, by which the HIV genome mutations confer lower sensitivity to one or more drugs, have been associated with the presence of LLV [34–41]. However, there is conflicting data with regards to LLV and the family of ART drugs used, mainly protease inhibitors (PIs), non-analogue reverse transcriptase inhibitors (NNRTI), integrase inhibitors (INIs). While Konstantopoulus (2015) found an increased risk of LLV in patients taking PIs compared to those taking NNRTI [34], later studies have not confirmed these data, probably because treatment based on PIs or NNRTI is usually prescribed in patients with more advanced disease or with adherence problems [42]. Moreover, high amounts of HIV-RNA in cells have been also observed in pLLV patients [35].

ART scale-up has improved quality of life for many HIV patients, preventing AIDS deaths and reducing new HIV infections. However, many patients develop HIV drug resistance (HIVDR) due to one or more mutations in the genetic structure of HIV that prevents the blocking of virus replication by a specific drug or a combination of drugs [43,44]. The impact of these HIVDR has been also assessed in pLLV patients [11,15,39–41,45,46] (Table 2). Swenson’s group (2014) considered that the presence of drug resistance mutations could predict VF in pLLV patients [16], and a direct association between HIV drug resistance mutations and ART was identified in viral RNA in pLLV patients [11,15,39,40,45,46]. Moreover, an elevated rate of mutations in the proviral DNA of pLLV patients has been observed [41], with this rate directly proportional to the viral load [46] (Table 2). Mutations in proviral DNA could play an important role in the prognosis of patients, without the need to be present in viruses with replicative capacity. Indeed, switching therapy in patients without a fully susceptible virus has been reported to entail an increased risk of virological rebound [47].

**Table 2.** Resistance associated mutations (RAM) detected in patients with pLLV

STUDY	YEAR	VL (cp/mL)	N_failure_/N_total_ (%)	NRTI	NNRTI	PI	II
**[**46**]**	2010	< 300	270/449 (60)	M41L D67N K70R L210W T215F K219Q/E M184 V K65R L74V	K103N Y181C G190A V1081	L90M M461 V82A D30N	NA
		300–999	399/552 (72)				
**[**11**]**	2011	50–1000	20/54 (37)	M184V A62A/V D67D/N K70K/P V75I	M230L/M K103N K101E V106M V106I Y188C/Y P225H/P V108I	D30D/N	NA
**[**15**]**	2015	50-500	11/48 (23)	M184V/I K219E L74V D67N L210W T215Y	L103N	L10F/I L33F/V I53L L63P I47V L76V G48V A71V V77I V82T I84V	T97A N155H T97A Y143C G163R
**[**39**]**	2013	50–1999	UNS	M184V	K103N Y188C	D30N L90M	NA
**[**45**]**	2011	50–200	209/396 (52.8)	UNS	UNS	UNS	NA
		201–500	201/287 (70)				
		501–1000	179/242 (74)				
**[**41**]**	2020	20–500	11/16 (68.8)	T215L/S D67DN/DE	V106I E138EA K103NS V179VD M230MI	K43T T74TP	G163R/K E138K L33LF E157Q G163R

Notes: pLLV: Persistent low-level Viremia; VL: Viral load; NRTI: Nucleoside reverse transcriptase inhibitors; NNRTI: Non-nucleoside reverse transcriptase inhibitors; PI: Protease inhibitors; II: Integrase inhibitors; UNS: Unspecified; NA: Not applicable. *Patients which LV was below to 500cp/mL was undetectable by sequencing.

By contrast, other studies have not shown any association between pLLV and viral resistance or inadequate drug concentrations [48,49]. Pereira et al. (2019) found no association between VF and the presence of HIVDR in pLLV patients [38]. While the presence of HIVDR hinders the ability to achieve undetectable viral load (uVL), it does not appear to be responsible for virological rebound events observed after virological suppression [50].

The **intensification of ARV is not associated** with a reduction in the incidence of pLLV in PWLH. Two studies that intensify ART with Raltegravir in patients with pLLV found no benefit when compared to control patients (without Raltegravir) [51,52].

By contrast, the optimization of ART related with a **switch to a second-line ART** based on PI may improve virological suppression (<50cp/mL) in a high percentage of pLLV (from 55% to 83.3%) [40,53–56] although the switch to Dolutegravir-based therapy has not shown a lower risk of developing LLV compared to other PI-based therapies [57].

Given that most published data belong to the pre-integrase inhibitors (INIs) era, the potential role of INIs as a treatment backbone remains unclear in the clinical management of pLLV. According to Taramasso et al. (2020), INI-based therapies could lead to lower pLLV over time and therefore to the achievement of more effective virological control [40]. More studies related to the use of INIs in pLLV patients are needed for more evidence-based data.

Finally, the clinical impact of pLLV on **mortality and AIDS events** also remains uncertain (Table 3). Some results have shown a risk of mortality or AIDS events that is 2–3 times higher [30,58], while others have not confirmed this [27,32,59–61] (Table 3). In Eastburn’s study, an association between mortality and RNA-levels was not observed, despite trying to adjust for different confounding variables such as cardiovascular risk factors and inflammation (OR: 0.99, p = 0.90) or duration of treatment (OR: 1.01, p = 0.91) [61].

**Table 3.** Published studies regarding different clinical consequences in pLLV *vs*. non-pLLV patients

YEAR	STUDY	HIV Patients (cp/mL)	n	Follow up (years)	VF		MORTALITY			AIDS EVENTS		NADES				IMMUNE ACTIVATION			
												GLOBAL		D-DIMER MARKER		IL-6		MICROBIAL TRANSLOCATION	
					aHR 95% (CI)	p	aHR 95% (CI)		p	aHR 95% (CI)	P	aHR 95% (IC)	p	t-test 95% (CI)	p	Statistics 95% (CI)	p	Statistics 95% (CI)	p
2020	**[**58**]**	No pLLV (<50)	4177	7.5	NA		1(Reference)		<0.05	NA		1(Reference)	-	NA		NA		NA	
		pLLV (50–199)	339				2.2 (1.3–3.8)					0.86 (0.50–1.5)	NS						
		pLLV (200–999)	258				2.1 (0.96–4.7)					2.0 (1.2–3.6)	<0.05						
2019	**[**62**]**	no pLLV (<50)	34	1	NA		NA			NA		NA		756 (157–3626)	0.038	NA		t-test = 1.30 (0.59–2.84)	0.62
		pLLV (50–999)	34											1114 (125–9917)				t-test = 1.23 (0.56–2.70)	
2019	**[**28**]**	No pLLV (<50)	2533	10	1 (Reference)	-	NA			NA		NA		NA		NA		NA	
		pLLV (51–200)	556		1.83 (1.10–3.04)	<0.05													
		pLLV (201–500)	110		4.26 (2.65–6.86)	<0.05													
2019	**[**25**]**	No pLLV (<50)	1090	21.8	1 (Reference)	-	NA			NA		NA		NA		NA		NA	
		iLLV (50–199)	374		0.33 (0.21–0.52)	<0.0001													
		pLLV (50–199)	150		3.46 (2.42–4.93)	<0.0001													
		hLV (200–1000)	392		2.29 (1.78–2.96)	<0.0001													
2018	**[**30**]**	No pLLV (<50)	5581	11	1(Reference)	<0.001	Analized with AIDS events.			1(Reference)	0.01	1 (Reference)	0.81	NA		NA		NA	
		pLLV (50–199)	237		1.42 (0.70–2.58)					1.44 (0.69–3.03)		0.81 (0.37–1.75)							
		pLLL (200–499)	168		3.25 (1.77–5.99)					2.89 (1.41–5.92)		0.83 (0.34–2.97)							
2017	**[**32**]**	No pLLV (<50)	716	20	1 (Reference)	-	1 (Reference)		-	NA		NA		NA		NA		NA	
		pLLV (50–199)	46		1.01 (0.23–4.31)	0.99	2.19 (0.90–5.37)		0.09										
		pLLV (200–999)	52		3.14 (1.17–7.03)	<0.01	2.29 (0.98–5.32)		0.05										
2017	**[**71**]**	No pLLV (<50)	113	4	NA		NA			NA		NA		NA		OR = 0.99 (0.96–1.02)	0.386	OR = 1.66 (1.32–2.08)	<0.001
		pLLV (50–200)	95																
2015	**[**24**]**	No pLLV (<50)	2169	11	1 (Reference)	<0.001	NA			NA		NA		NA		NA		NA	
		pLLV (50–199)	205		2.30 (1.66–3.20)														
2015	**[**27**]**	No pLLV (<50)	16,796	2.7	1 (Reference)	<0.001	Analized with AIDS events.			1 (Reference)	NS	NA		NA		NA		NA	
		pLLV (50–199)	624		1.38 (0.96–2.00)					1.19 (0.78–1.82)									
		pLLV (200–499)	482		3.97 (3.05–5.17)					1.11 (0.72–1.71)									
2013	**[**63**]**	No pLLV (<20)	39	UNS	NA		NA			NA		NA		NA		MED = 79 (61–105)	<0.05	MED = 4.1 (2.8–6.1)	NS
		pLLV (20–200)	13													MED = 90 (81–154)		MED = 6.1 (2.8–9.3)	
2012	**[**73**]**	Healthy	15	UNS	NA		NA			NA		NA		NA		Rho = 0.217	0.017	Rho = −0.015	0.870
		pLLV (1–500)	122																
2011	**[**61**]**	No pLLV (0)	210	5	NA		OR:0.99*	0.90		NA		NA		NA		1 (Reference)	-	NA	
		pLLV (1–19)	137													LLM = 0.1 (−26, 35.5)	0.99		
		pLLV(20–399)	236				OR: 1.01**	0.91								LLM = 0.7 (−23.5, 32.6)	0.96		
		pLLV (400–1000)	237													LLM = −2.5 (−45.6, 74.8)	0.93		

## Inmunological activation and inflammation status in pllv patients

An association between HIV chronicity and increased inflammation and immune system activation has been established previously [62–65], with the maintenance of suppressed viremia thus remaining essential. Although the use of ART serves to remarkably decrease both inflammation and activation in PLWH, complete restoration of the immune system remains elusive due to the presence of residual persistent inflammation [66–69].

Persistent low-level viremia may also affect immune activation and inflammation status. However, discordant results published thus far prevent us from gaining a general overview of its real impact.

Regarding **immune activation** status, a significantly elevated **immune activation**, defined as CD38+ HLA-DR+ in peripheral blood mononuclear cells, has been observed in pLLV individuals when compared to virologically suppressed subjects [14], in line with recent findings that identified specifically higher percentages of CD8+ HLA-DR+ and CD8+ CD38high T cells in pLLV subjects that lead to an excessive immune activation in adolescents and young adults [70].

In relation to **inflammation status**, an elevated risk of immunovirological failure and an increase in proinflammatory cytokine levels is still observed in pLLV subjects, unlike those with uVL [27,30,31,63]. The continuous presence of virus in pLLV patients could complicate the recovery of normal values related to **inflammatory biomarkers** [71] such as Interleukin 6 (IL-6), associated with higher levels in PLWH in contrast to healthy individuals [68,72]. However, the role of IL-6 in pLLV patients and its association with the degree of inflammation remains elusive (Table 3) because, although low IL-6 levels have been associated with pLWH with VL [73], other studies have not observed higher IL-6 levels in pLLV individuals compared to suppressed HIV viral load [61,63,71] (Table 3).

Microbial translocation (MT) entails the movement of commensal microbial products from intestinal lumen into circulation in the absence of bacteremia. In 2006, this mechanism was described for the first time in PWLH [74]. HIV infection causes important and irreversible intestinal damage, regardless of the route of transmission [75]. The presence of microbial products in circulation contributes to immune activation in PWLH [76,77]. MT has also been implicated in the degree of inflammation and immune activation present in pLLV patients [63,71]. Reus et al. (2013) showed less frequent MT (16S ribosomal DNA) in treated PLWH with uVL (20 copies/mL), in contrast to those with pLLV (20–200 copies/mL) [63]. Falasca et al. (2017) considered that MT could be the mechanism which leads to increased inflammation in pLLV patients compared to patients with undetectable VL [71]. The fact they showed a lack of virologic suppression during follow-up seems to be related to elevated levels of sCD14, a biomarker of MT [71] (Table 3).

## Non-aids defining events (nades) and PLLV

While AIDS events are no longer the principal problem in treated PLWH, new complications called Non-AIDs-Defining Events (NADEs) have become the main cause of morbi-mortality [78–84]. NADEs are determined by multiple factors such as age [85–89], immune status [90,91], treatment toxicity [92,93] and even lifestyle [86,89,94], which would explain the relationship between NADEs and increased morbi-mortality. ART allows patients to live longer but does not completely restore their immune system, leading to the appearance of cardiovascular diseases and cancer, among others diseases [95–98]. Moreover, as a consequence of the chronification of the disease, long-term exposure to these drugs appears to be implicated in metabolic and organ damage [99–106]. However, the new ART families are increasingly safe and tolerable, with metabolic damage most likely caused by the HIV itself rather than by the drug’s toxicity [107]. The occurrence of NADES are closely associated with inflammation and immune activation [61,62,68] and it has been suggested that LLV phenomena which lead to residual immune activation and inflammation states may influence the morbidity and mortality of NADEs [32,73,108,109].

The main studies on NADEs have been related to cardiovascular diseases, where increases in C-reactive protein (CRP) and D-dimer biomarkers have been found in PLWH compared to healthy controls [81,110]. However, the potential role of pLLV in the emergence of NADES remains unclear. PLLV patients presented levels of D-dimer that are 1.5 times higher than persistent virologic suppressed individuals [P = 0.038], which may increase the risk of developing cardiovascular diseases [62]. This stands in contrast with the lack of association observed between CRP levels and low-level viremia [61]. Yet other results have shown no association [30,58,61]. In the Spanish AIDS Research Network cohort (CoRIS), pLLV (between 200 and 499 copies/mL) was associated with virological failure and AIDS events/mortality, but not with NADEs [30]. The Swedish Nationwide Observational Study also showed that pLLV between 50 and 999 copies/mL was related to mortality, but not with a higher risk of severe NADEs or AIDS [58] (Table 3). However, a subanalysis of this study with pLLV between 200 and 999 copies/mL demonstrated a two-fold increase in the probability of suffering severe NADES [58] (Table 3).

Overall, no consensus has been reached as to the clinical consequences of pLLV. While some studies claim that persistent LLV increases the risk of future virological failure, others have not even found a significant impact on AIDS events and mortality. While the worst impacts may be associated with VL above 200 cp/ml, it is less clear if there is any negative impact on virological failure and morbi-mortality when LLV is between 50 and 200 copies. Different methodologies and a lack of standardization in the number and characteristics of populations studied make it difficult to compare results (Table 3). Further studies are needed to find new evidence for the wellbeing of patients and to identify long-term outcomes in LLV individuals.

### Origin of pLLV: THE relationship with viral reservoir and clonal expansion

Most studies published suggest that factors such as ART adherence difficulties and viral genotype resistance could be the main cause of pLLV in HIV patients, as we have previously reviewed [34,36–38,40,50]. However, although pLLV status does not seem to be a random biological phenomenon [54], the origin of this phenomenon remains unclear and the source(s) and mechanism(s) continue to be largely undescribed. Viral reservoir size [20,111–114] and clonal expansion [115–117] could be also linked to their origin.

**The HIV viral reservoir** is the main barrier to curing HIV infection [118–120]. HIV reservoirs are formed very early at the onset of infection and ART currently remains unavailable [119,121–124]. Although HIV reservoirs tend to decline slowly over time [125], the influence of persistent immune pressure on the evolution of reservoir size during ART should be considered [126]. Indeed, the decrease observed in proviral DNA sequences linked to this HIV reservoir decline is variable and difficult to determine in patients with a weakened immune system [127–129].

Despite the unknown origins of low-level viremia, in 2015, Samarti et al. [130] proposed two possible hypotheses related on the correlation of pLLV and HIV reservoir: the filling of reservoirs as a consequence of ongoing HIV viral replication; and the emptying of reservoirs during effective ART. No conclusions were reached due to a lack of studies and limited data, however.

More recently, Jacobs et al. (2019) reviewed the issue and found a generally direct association between HIV-DNA levels and persistent viremia, despite the differences observed regarding techniques used and findings [131]. This could explain why viral reservoir size and the emergence of pLLV were not correlated by Widera et al. (2017) [132]. Moreover, from the studies reviewed, Jacobs et al. were unable to identify a cellular or titular reservoir contributing to persistent viremia and thus proposed proviral replication as the main cause of the continuous presence of the virus [133].

**Clonal expansion** also complicates HIV clearance and contributes to the maintenance of reservoirs [126], probably through viral splicing, integration into oncogenes, and contributing to the expansion of proviral HIV clones [134–137]. This relationship has been demonstrated by several studies [136,138,139] and we hypothesize as to the possibility that both mechanisms may be associated with the emergence of pLLV in PLWH [140]. This hypothesis has been demonstrated by Pinzone et al. (2019), who observed that one of their patients who had maintained an LLV status for 9 years had intact HIV clone sequences [126]. This would be supported by studies carried out in HIV patients where the proliferation of cells with proviruses was also suggested as a mechanism of viral persistence [141,142]. According to this result, other studies have found a large viral reservoir size quantified through HIV-DNA levels in patients receiving effective ART treatment with residual viremia associated with blips [143,144]. Indeed, LLV and blips have been closely associated with a slow elimination of the viral reservoir [145].

Determining the role of the viral reservoir and the mechanism of clonal expansion in PLWH with pLLV remains crucial for the design of therapies that achieve HIV elimination (54).

## Concluding remarks

Further knowledge of the continuous presence of the virus in treated PLWH is needed to design treatments that achieve a cure for HIV infection. Reported data regarding the possible causes and origins of pLLV and its impact on clinical progression are rather discordant. While some studies point to a higher risk of developing virological failure and different AIDS/mortality events related to pLLV status, others have not made such findings. Moreover, the emergence of viral resistance in pLLV patients and its direct association with ART adherence have been only partially observed (Table 4).

**Table 4.** Summary of the role of pLLV

SUMMARY: ROLE OF PLLV		
CLINICAL IMPLICATIONS		
Increased risk of developing VF		[24,25,27,28,30–33]
Nature of ART and adherence difficulties		[34–37]
Drug resistance mutations	Association	[11,15,16,39–41,46]
	Non association	[38,48–50]
ART intensification with Raltegravir not reducing pLLV		[51,52]
A second-line ART improving pLLV status		[40,53–56]
A second-line ART not improving pLLV status		[57]
MORTALITY AND AIDS EVENTS		
Higher risk of mortality and AIDS events		[30,58]
No higher risk of mortality and AIDS events		[27,32,59–61]
IMMUNOLOGICAL ACTIVATION AND INFLAMMATION STATUS		
Immune activation status		[14,70]
IL-6 levels	Association	[73]
	Non association	[61,63,71]
Relationship with MT (elevated levels of sCD14)		[63,71]
NON-AIDS DEFINING EVENTS (NADES)		
Possible influence in the morbidity and mortality of NADES		[32,73,108,109]
Relationship with cardiovascular diseases		[58,62]
No association with NADES		[30,61]
ORIGIN OF PLLV		
Viral reservoir		[130,131,133]
Relationship between viral reservoir and clonal expansion		[126,140–142]

Notes: pLLV: Persistent low-level patients, VF: Virological failure; ART: Antiretroviral treatment; AIDS: Acquired immunodeficiency syndrome; MT: Microbiotal translocation; NADES: Non-AIDS-defining events.

On the other hand, patients with pLLV seem to have an elevated risk of immunovirological failure and increased levels of proinflammatory cytokines, and there seems to be a general consensus as to the association of pLLV with persistent immune system activation. Indeed, it has been speculated that the phenomenon of pLLV, which leads to residual immune activation and inflammation, may influence the morbidity and mortality of NADEs.

Related to the origin of this phenomenon, the clonal expansion in HIV-infected cells and its impact on the maintenance of the viral reservoir have been previously described and may be related to the appearance of pLLV in treated HIV patients (Table 4). A lack of studies and standardization make further studies necessary to clarify the origins and causes and in which a uniform definition of pLLV status should be established.

## Data Availability

The data of this study are available from the corresponding author [VB].
